# Bridging Formal Health Services and Community Care for Improved Access to Culturally Appropriate End-of-Life Support for Migrant Communities

**DOI:** 10.3390/ijerph23070888

**Published:** 2026-07-10

**Authors:** Rosemary Leonard, Joy Paton, Peta Hinton

**Affiliations:** 1 School of Social Sciences, Western Sydney University, Locked Bag 1797, Penrith, NSW 2751, Australia; joy.paton@westernsydney.edu.au (J.P.); p.hinton@westernsydney.edu.au (P.H.); 2 Translational Health Research Institute, Western Sydney University, Locked Bag 1797, Penrith, NSW 2751, Australia

**Keywords:** migrant communities, palliative care, death literacy, community services

## Abstract

**Highlights:**

**Public health relevance—How does this work relate to a public health issue?**

- The World Health Organization has identified access to palliative care as a global ethical responsibility and the end-of-life care needs of migrants have been identified internationally as an area of specific concern.
- Recognition of end of life as a social and cultural event, in which medical treatment is just one component of care, is needed to enable carers and patients to focus on physical and emotional comfort, making the end-of-life path less distressing for the dying person, their family and community.

**Public health significance—Why is this work of significance to public health?**

- The research highlights different cultural needs at end of life for the migrant populations involved in the study and shows some of the ways that end-of-life care can be provided in culturally safe ways, informing both practice and policy in palliative care and bereavement service delivery.
- Barriers to good end-of-life care include institutional ‘silos’ in the medical and social welfare systems, alongside language and communication barriers including appropriate ways of talking about end of life, and unmet cultural needs including the capacity to follow traditional rituals and decision-making practices.

**Public health implications—What are the key implications or messages for practitioners, policy makers and/or researchers in public health?**

- A range of existing resources can be utilised and enhanced, alongside the development of innovative service delivery supports, for overcoming barriers to accessing culturally appropriate end-of-life and bereavement support for migrant communities.
- Building cultural sensitivity, knowledge and humility into practice and policy is foundational for progressing changes to meet palliative care and bereavement needs and for expanding research in this space that includes the voices of migrants as the people for whom services are developed.

**Abstract:**

The research aimed to understand the different cultural needs relating to death and dying of migrant populations in Western Sydney, Australia, and how end-of-life care can be provided in culturally safe ways which can, in turn, inform the policies that shape palliative care and bereavement service delivery. The mixed methods design for this study used an online quantitative survey using the Death Literacy Index, key informant qualitative interviews, culture-based community focus groups, and individual Photovoice interviews with carers. In all, 266 participants across the three largest migrant communities and local area health services took part in the research. The results revealed the importance of cultural practices and rituals for people at end of life and that providing space for these is crucial to cultural safety in service contexts, in addition to relational trust and the need for community input to improve the services that affect them. There is also a need for greater knowledge and understanding in the end-of-life space through two-way exchanges between communities and service providers. Finally, there are important ways that the existing services can facilitate cultural safety and ways of increasing the availability of culturally appropriate end-of-life and bereavement services for the community.

## 1. Introduction

Healthcare systems serve the living and the dying. Palliative care strives to improve the quality of life for people living with life-limiting illness, as well as their caregivers. It is “person- and family-centred care” that aims to ensure death with comfort and dignity, supported by high-quality care that aligns with the person’s values and preferences [1]. The World Health Organization has identified access to palliative care as a global ethical responsibility, defining it as “a crucial part of integrated, people-centred health services” [2]. Strengthening equitable access to palliative care services is a WHO priority and is integral to its goal of universal health coverage under the United Nations [3] Sustainable Development Goals (SDG 3). Furthermore, the palliative care needs of migrants have been identified internationally as an area of specific concern, especially in terms of capacity in the local language used for crucial communications around disease, prognosis, treatment, and care preferences during the end-of-life (EOL) phase [4,5,6]. The latter “typically refers to the 12 months prior to death” of someone facing a life-limiting illness [7] (p. 1).

However, formal health services do not function in isolation from families and communities and are not always the principal care preference for people when they have a life-limiting illness. Horsfall et al. [8] identified the importance of home as a place of caring and, in the Australian context, demonstrated that the majority of EOL caring in the general population occurs at home, even when there are multiple trips to the hospital, because of the many benefits for the dying and their carers. These include being surrounded by memorabilia and artefacts; familiar food and music; contact with friends, neighbours, cultural or religious community; ability to enjoy social events; and being close to one’s garden, pets, and nature [8]. Home also provides places for carers to have time out; reduces government health service costs; and provides non-threatening opportunities for others to learn about EOL care. These benefits of home might be even more important to migrants from different cultures for whom Australian hospitals and Aged Care facilities can be very unfamiliar and intimidating environments.

EOL care spanning public systems, community networks and familial spaces is supported by a complex system of paid and unpaid services [9]. Palliative care nurses and physicians and palliative care volunteers work in hospitals, hospices, and ‘the hospital in the home’ (medical treatment delivered in the patient’s home). Beyond this, numerous other services and supports include community nurses, general practitioners (GPs), homecare and in-home respite services, practical home help for carers, community transport, disease specific family support workers, death doulas, community mentors, grief and bereavement counsellors and disease-specific support groups. However, such complex arrangements also have the potential for significant problems, especially for EOL care in the home. Rosenberg et al. [9] noted that these include the absence of home visits by doctors, lack of 24 h support, and insufficient GP referrals of people at EOL to community and palliative services. Lack of overall coordination of services and flow of information about services were identified as being important contributing factors [9].

The difficulties identified above for the general population raise concerns for Culturally and Linguistically Diverse (CaLD) people. Language differences, cultural norms and unfamiliar health systems are factors that potentially impact the capacity for access and navigate EOL care and services. This highlights the importance of a research focus that is specifically designed for migrant communities that takes account of the spaces within and outside of formal health services and investigates the needs of migrant communities across the spectrum of EOL services. In this article, we present findings about the EOL and bereavement needs in (selected) migrant communities, drawing on research [10] conducted within the Western Sydney Local Health District (WSLHD), a large state-based health district in Sydney, Australia. Below, we provide relevant background information before outlining the multi-method research design and approach to data analysis. Following this, we discuss the results of the research and strategies for overcoming barriers to good EOL care for migrant populations. We address the potential limitations of the design before providing concluding comments.

## 2. Background: EOL and Migrant Populations in Australia

Following the dominance of British migration resulting from colonisation some 250 years ago, there have been waves of migrants from other cultures prompted by opportunities in Australia, or by war and hardship in their country of origin. The Australian government has actively encouraged migrants as a source of economic growth and to meet humanitarian commitments. Yet, in reviewing government policies in the context of refugees, Fozdar & Banki [11] highlighted the lengthy waiting times (often many years) to qualify for government health and social support, as well as temporary visa rules whereby migrants cannot leave the country, else they risk losing the chance to become Australian citizens. As a result, extreme disconnection can be experienced. In addition to complex processes for accessing services, migrants, including non-refugees, can have histories of hardship or trauma due to poor governance and services, or even violence. On arrival, they can experience separation from their known social world and culture shock in the new world (see for example [12]).

Because of lengthy waiting periods for receiving government health and social support, an income stream is essential. Australian health services are based on a liberal individualist philosophy which assumes that the best way to deliver services is to construct people as consumers who choose the services they need and decide if they wish to pay directly for private provision or wait for free public services [13]. Groups marginalised by language or culture effectively have restricted access to healthcare services and often suffer poorer health as a result [14]. Access to healthcare is further impeded by the economic stress that is sometimes caused by a lack of recognition of overseas credentials, which can lead to long hours in low-paid and insecure work [11].

Despite being a ‘multicultural’ society, overt racism is also present. This may occur, for example, in places of disadvantage, where existing residents sometimes resent the ‘competition’ from new arrivals [15]. However, government policies are also implicated here. For example, during the COVID-19 pandemic, the Australian Government allowed British heritage Australians to return from the U.K. but did not allow Indian heritage Australians to return from India [16]. The impacts of this policy were felt acutely by the Hindi population living in Western Sydney who were participating in our research.

CaLD populations are a significant proportion of the people living in Western Sydney. The current Australian Bureau of Statistics [17] census data indicate a population of 1,108,820 people, and a lower median age (35 years) than the state of New South Wales (39 years), which reflects inward migration to the region. Within WSLHD, 58.3% of people are non-English speakers at home. The three largest migrant groups are people from Mandarin- (6.5%), Hindi- (3.5%), and Arabic (5.3%)-speaking backgrounds. Although substantial in comparative numbers, CaLD populations in WSLHD are nevertheless ‘marginalised’, culturally and economically. Large areas of the LHD have populations with low average incomes, so few can afford private care at EOL. Furthermore, cultural marginalisation, while evident in healthcare settings generally, is especially prevalent in EOL [18,19].

Language barriers have been identified as an issue of marginalisation in relation to (medical) communications about disease and EOL preferences [4]. However, there is a concomitant issue with regard to the discourse itself. Patients and caregivers assume that at EOL, the ‘real discourse’ is about the disease. Healthcare is hindered by insufficient recognition of the patient as a person dwelling in a particular lifeworld, in embodied, social, sentient, and skilful ways. This causes people to overlook the equally real, lived experience of the patient, which requires a different descriptive discourse and different sources of understanding from the medical discourse. A lack of dialogue between these two discourses has negative effects on patients because caregivers need both in order to provide attuned, effective caregiving [20].

Migrant populations are especially vulnerable to this discourse problem, which is exacerbated when people’s EOL beliefs and practices conflict with dominant ‘mainstream’ expectations. An alternative perspective is to construct death and dying as being essentially social, rather than solely medical, events [21]. Health Promoting Palliative Care [22] emphasises trusting relationships and networks of care across health services and communities. This new public health approach to EOL care is encapsulated in the Compassionate Communities (CC) movement [22]. In supporting people to manage EOL, CCs acknowledge isolation, burden, anxiety, and fear, which are understood as social problems that are best addressed by a whole-of-community approach [23,24].

One way of examining people’s ability to manage EOL is through the concept of death literacy, defined as “a set of knowledge and skills that make it possible to…understand and act upon end-of-life and death care options” [25] (p. 32). The Death Literacy Index (DLI), initially developed in Australia, measures knowledge, skills, experiential learning and social action, which reflect people’s capacity to access, understand, and make informed choices about EOL care options [26]. People and communities with high levels of death literacy have context-specific knowledge about death systems and the ability to put that knowledge into practice. Analysis of a Nationally representative survey shows that people from non-English speaking backgrounds, including the three language groups in this study, have significantly lower DLI scores [26]. Low death literacy is reflected in a lack of appropriate EOL services.

Indeed, there is a schism between the Australian National Palliative Care Strategy 2018 that demands that high quality evidence-based palliative care is accessible to all Australians, and the Department of Health [27] data showing that CaLD Australians are currently under-served. Access to formal palliative care services is under-utilised within the general population [28], and more so in CaLD and migrant communities [10]. The relative marginality of formal health and welfare services has been demonstrated in previous research using social network analysis of EOL caring networks, which showed that these are dominated by family and friends [29]. Within such networks, having existing professional contacts or a relative in the health field made it easier to navigate systems and obtain needed help [29]. Without such contacts, engaging with formal EOL service providers was challenging and the systems were difficult to navigate. Access challenges for the general population and the reality of unmet servicing for CaLD people at EOL, as acknowledged by the Department of Health [27], informed the design of research outlined below to better understand the EOL needs of migrant communities within WSLHD.

## 3. Research Design: The EOL Needs of Migrant Populations

The research aimed to understand EOL needs for people from Hindi-, Arabic- and Mandarin-speaking backgrounds in the Western Sydney Local Health District (WSLHD). It was designed to engage directly with communities, giving a voice to those who are marginalised, vulnerable, or disadvantaged. The research was conducted by Western Sydney University’s Caring at End-of-Life Research Program and supported by a WSLHD Advisory Panel, including representatives from Supportive and Palliative Care and Multicultural Health. Consultations with individual cultural advisors from local communities were also undertaken as part of the research design process. Through amplifying marginalised voices, the research aimed to better understand cultural needs relating to death, dying and bereavement and how culturally safe services can be provided for people at EOL and those who care for them.

Death is recognised as a challenging research topic [30,31] and this project had the added complexity of cultural considerations. Acknowledging that people are the ‘experts’ on their own experience [32], we sought to develop relationships of equality and to ensure that the exchange of knowledge between researchers and participants met the research goals in ways that were simultaneously culturally sensitive. The principal of cultural humility [33] underpinned both our engagement with participants and consultations with Cultural Advisors and Multicultural Health (MCH) Education Officers. The researchers are indebted to these people and the WSLHD Advisory Panel, who created a ‘bridge’ for us to conduct the research. As trusted community contacts, their familiarity with the target populations helped with the design process and was invaluable in recruitment efforts, especially for EOL carers across the three language groups. They also identified appropriate (safe) community spaces to conduct face-to-face research activities. It was a privilege to do this research and the care taken in thinking through the research design and the ways it would be implemented are evident, we believe, in the excellent participant retention rate for all methods of data collection (nil withdrawals from the study).

### 3.1. Methods for Data Collection

The research engaged healthcare professionals and community members in a comprehensive multi-method project to elicit both qualitative and quantitative data. Qualitative elements designed to hear directly from people using or working in EOL services comprised key informant interviews, community focus groups, and individual photovoice interviews. An online survey, based on the National Death Literacy Index (see [26]), was used to establish a numerical understanding of death literacy within the LHD, and by implication, people’s capacity to manage at EOL. These four methods are outlined below, along with the recruitment processes and details of participants (NOTE: a formal collection of the demographic data was not undertaken, as the Advisory Panel and community cultural advisors counselled that this was likely to be considered intrusive by most participants. Gender and age approximations were collected by researcher observation and as declared by respondents to the online survey).

#### 3.1.1. Key Informant (KI) Interviews

Individuals with relevant knowledge of, and experience working in, EOL and bereavement services were recruited for the KI interviews. The interview schedule asked participants to: (1) describe their roles and experience with the three migrant communities; (2) share their knowledge of the traditions around death, dying and caring and any observed differences compared to ‘mainstream’ populations; (3) outline existing services and their ideas for improvements; and (4) describe additional services they would recommend. The 2 h interviews were recorded on Zoom.

Participants: These were adults (43), primarily healthcare workers and professionals within supportive, generalist or palliative EOL care, who worked within WSLHD, but also included some adult people working in EOL or bereavement-related services outside the LHD. The KIs represented a range of cultural backgrounds, including the three target groups (Arabic 5; Hindi 7; Mandarin 4; Other 27. Gender: M 8; F 35).

Recruitment: Initial contacts provided by the WSLHD Advisory Panel and Multicultural Health Education Officers were invited by the research team to participate and to make suggestions for additional KIs. The spread of interviewees was reviewed to identify gaps (e.g., general practitioners) which were subsequently filled. KIs were recruited to saturate themes. This ongoing purposive (and limited snowballing) recruitment ensured that KIs were people with relevant expertise and specific knowledge of WSLHD (or similar LHD).

#### 3.1.2. Community Focus Groups (FG)

Language-based FGs with community members were utilised to gather data about the cultural differences and needs at EOL (see [34]). FGs also provided a forum to explore possible barriers, strategies or opportunities for developing culturally sensitive EOL and bereavement services. Separate face-to-face FGs for each migrant group were conducted at culturally appropriate community centres with dates adjusted around COVID-19 lockdowns. The FGs were cofacilitated by a member of the research team and a bilingual Multicultural Health (MCH) Education Officer sharing the language and cultural background of group participants. A State-accredited translator was also present. The Hindi community focus group was conducted in English, at the request of the participants (this was not unexpected, given that both English and Hindi are official languages of India). Following verbal then written consent, participants in each focus group were offered opportunities for opting out during the audio-recorded 2 h sessions, in line with the process consent method (see for example [35]). All participants completed the process, which was audio-recorded and transcribed.

Participants: These were adult community members (33) living in WSLHD who had cared for someone at EOL (in a personal, not professional capacity) for more than six months prior to the research (Arabic 13; Mandarin 10; Hindi 10. Gender: M 15; F 18). The three language-based groups comprised community members who attended MCH education programmes within WSLHD.

Recruitment: Digital and printed recruitment flyers were distributed in English, Mandarin, Arabic and Hindi by community representatives, MCH and WSLHD networks. Interested individuals meeting the inclusion criteria were sent a participant information sheet. MCH education teams provided in-depth explanations about the research in the preferred language of potential participants, while assuring that participation would be voluntary and research material would be anonymous. FG numbers were limited by what was practical in size and the capacity of MCH partners to recruit people for a sensitive, and potentially culturally taboo, topic. With FG recruitment initiated through MCH, a government service with an outwardly focused community of workers who were interested in the diverse population of Western Sydney, participation was effectively limited to people who had previous contact with MCH. This marginalised users of private or community services outside the public health system who could have potentially contributed relevant data about EOL experiences. However, trusted MCH contacts and familiar community spaces were more likely to encourage participation in the research, especially of EOL carers.

#### 3.1.3. Photovoice (PV) Interviews

Photovoice (PV) is a helpful method in sensitive enquiry [32] because visual material such as photographs helps us to enter research conversations that can be personally challenging. In this project, we used PV with carers to learn more about what works (or not) in the health service system and wider community to support people who are caring for someone at EOL. The method was modified to work online, within a context of community lockdowns due to COVID-19. Semi-structured, recorded and transcribed online interviews of up to two hours were held with individual participants. All interviews were conducted by a researcher speaking English with the option for a nominated bilingual support person to assist with translation as needed. The discussions focused on experiences of care prompted by the photographs and were guided by a series of questions based on project information previously given to participants. An alias was chosen to protect anonymity in the dissemination of research findings.

Participants: These were adult community members (11) living in WSLHD and who were a personal carer for someone at EOL for more than six months prior to the research (Arabic 4; Hindi 3; Mandarin 4. Gender: M 2; F 9).

Recruitment: Recruitment of up to four PV participants for each language group was primarily conducted by MCH Project Officers alongside the distribution of electronic and print flyers in English, Mandarin, Arabic and Hindi throughout the LHD. Additionally, the online Death Literacy Survey invited respondents to leave contact details if they wanted to know more about the PV process. People expressing interest were contacted by a researcher to discuss the PV method. Those meeting the selection criteria and wanting to participate were sent the project documents and consent form by post or email.

#### 3.1.4. Death Literacy Index (DLI) Online Survey

The DLI survey was accessed online through the WSLHD website (the URL was displayed on flyers with a QR code linking people’s smart phones to the webpage). The survey was available in English, Arabic, Hindi, and Mandarin. The introductory page informed respondents that their participation was anonymous and outlined the research aims along with what was involved in completing the survey. The survey tool was released in February 2021 and remained live online for a total of 8 months. This was longer than initially planned due to slower-than-expected uptake, most likely due to the COVID-19 pandemic.

Participants: There were 179 respondents across the three communities (Arabic 36; Hindi 60; Mandarin 83. Gender: M 61; F 116; Not identified 2). We noted that 40% of respondents were over 65 years of age due to recruitment and support for completing the survey being undertaken by MCH educators during business hours.

Recruitment: Recruitment was primarily conducted by members of MCH education teams, who approached their contacts within the community, along with the community members in the WSLHD health education programmes. The survey was also promoted through digital flyers that were forwarded to health professionals and community workers to circulate amongst their networks. Paper flyers and posters were distributed to 42 general practices and medical centres and five community centres throughout WSLHD.

### 3.2. Data Analysis

#### 3.2.1. Qualitative Data from Key Informant (KI), Focus Group (FG) and Photovoice (PV) Interviews

The interview transcripts were de-identified and analysed independently by at least two researchers. The Key Informant transcripts were analysed as one data set. The Photovoice transcripts were analysed as a collective data set, as well as individually, to distil a concise narrative for each participant. The Focus Group transcripts were analysed separately. Analysing transcripts was an interpretative, qualitative and data-driven inductive process. Data were first analysed for content according to the specific topics of the research questions, while also noting unanticipated/emergent themes. Second, the transcripts were analysed thematically [36] to identify common and recurring themes, similarities, and points of difference among the cultural groups.

We acknowledge the potential power differentials between different research participant groups. For example, key informants have a different status and knowledge set in relation to EOL and bereavement care than those who access services. However, both perspectives are crucial and present in the aggregation of thematic data, bringing relevant insights to the overall aim of service improvement. Direct participant quotes, used below in the Discussion, are identified by source group (KI, FG, PV).

#### 3.2.2. Quantitative Data from the Death Literacy Index (DLI) Online Survey

Descriptive statistics were generated from the quantitative data obtained through the survey and these were further analysed using Version 29 of the IBM SPSS Statistical Package. Scores for the total DLI and its subscales were calculated on scales from 1 to 10, where 10 would indicate a very high level of death literacy [26]. The research question on demographic differences and differences in life experience was addressed through a series of linear models, with sociocultural group and the demographic variables as the predictor variables and the DLI as the dependent variable.

## 4. Results and Discussion

This section addresses key findings about the types of barriers faced by migrant populations at EOL and the ways that such barriers might be bridged. Some barriers impact the general population, too; but for the migrant experience, these become more impenetrable when fundamental institutional structures, forms of communication, and ‘taken-for-granted’ traditions are unfamiliar. Building bridges is important for patients, families, and carers, but also health workers and professionals trying to provide optimal care so that the EOL needs of migrant communities can be met in culturally safe ways.

### 4.1. Barriers to Good EOL Care

#### 4.1.1. Silos in the Medical and Social Welfare Systems

Previous research identified a separation between medical services and bereavement services, which often left carers with a sense of isolation or abandonment when the person they cared for died [8]. At times, formal service providers were seen as part of the community supporting the carer at home, so it could be a shock when the relationship ended abruptly upon the person’s death. A further ‘silo’ generating problems in the EOL space, which was particularly resonant for migrant populations, is the separation of health services from community organisations, religious organisations, and informal networks providing support in the community [8].

These various forms of institutional segmentation are consistent with the findings for WSLHD focused on migrant populations, with the addition of divisions among medical and social welfare systems. There was strong evidence of the medical and social service silos arising from a neoliberal mindset whereby people are conceived as customers choosing the service they want. The most extreme example was reported by a Hindi counsellor:

*One woman told me about seventeen services she had been to… And when you’re flung from one service to the other, mate, I would run away after the fifth service.*

(Counsellor, Hindi KI).

Within the hospital context in Australia, departments are determined by medical physiology, with each dealing with a different disease. Divisions between palliative care and other units can result in a lack of timely and appropriate referrals to palliative care. This is often because other health services are not aware of the support that palliative care provides or because *conversations are just not started early enough to say it is a life limiting illness* (Nurse, Community health KI).

A recently created silo is the outsourcing of palliative care in the home to a private provider. This initiative greatly increased the amount of care available, including a 24 h on-call doctor. However, palliative care staff in the hospital experienced insufficient communication at times, when patients had multiple hospital admissions and discharges. Furthermore, they expressed distress for themselves and for the patients at relational ruptures:

*We support them through their chemo, their radiation—the whole thing. And then right at the point where they’re literally dying, we have to say goodbye.*

(Community nurse, Hospital palliative care unit KI).

The private provider of in-home palliative care includes social workers in their service who could bridge the division between care for the family before and after the death. However, they often do not have the time to do so, due to heavy caseloads:

*Ideally, as a social worker, we’d like to take every client because how do you know whether someone needs grief and loss support just by their social circumstances? …Unfortunately, we don’t have capacity to take on a referral for every client that comes in. I have a client load of about fifty. So, to stay on top of all of that is stretching it. It’s tough.*

(Social worker, Palliative home care service KI).

While institutional silos are problematic for all service users, the impacts are more acute in the migrant context, where language and communication issues further alienate people from the health system.

#### 4.1.2. Language and Communication

Language barriers are the most obvious challenge for migrant populations from non-English speaking backgrounds and previous research has identified that communication barriers are the main problem in EOL [37]. Even with adequate English for everyday contexts, migrants can feel confused and isolated in the health systems, especially as acquired language can be more challenging during ageing and illness:

*In the nursing home he (father) felt himself alone. He couldn’t talk to people around him because of his background (heavy accent).*

(Indian Male, Hindi PV).

The language used by people working in hospitals and other health facilities is often unfamiliar, and a cumulative barrier for migrant families:

*[At a family conference with doctors] I couldn’t even understand what advance directives meant. I didn’t know those terminologies. They didn’t take the time to … make it easier for someone who doesn’t understand medical jargon.*

(Young Indian Female, Hindi PV).

Efforts to bridge the language barrier with brochures that include translations can inadvertently become barriers because they reinforce medical discourse which emphasises the ailment, rather than making a meaningful connection with patients:

*[Doctors] can now treat them just as a knee or a left foot because [they] know that—[they’ve] got a bit of paper that’s been translated.*

(Palliative care and bereavement counsellor KI).

Hospitals have the benefit of access to an expert, medically oriented interpreter service to enable effective communications. However, two difficulties arise. First, there is not always an equivalent available in the migrant’s language for certain ideas, concepts or words: *sometimes if there is no equivalent, we have to paraphrase. We double-check with the healthcare provider, “Is it okay if I say this?”* (Interpreter, Chinese KI). Second, there can be cultural barriers to mentioning death: *one interpreter… said “I am not going to translate ‘You are in a palliative care unit to die’, No!”* (Clinical nurse consultant, Community health KI).

Despite these difficulties, expert interpreter services are extremely important in the EOL context, yet community or private services, such as the home-based palliative care service, do not have access to them, unless they are privately funded.

#### 4.1.3. Unmet Cultural Needs of Migrant Populations

The difficulties that interpreters face when passing on the essential information needed for EOL planning and optimal care highlights the need to go beyond addressing language differences. It is also crucial to understand cultural differences and alternative needs that may be present for migrant patients, carers, and their families.

Data collected in the WSLHD DLI survey [10] allowed for a more in-depth exploration of issues facing migrant groups than the previous National Survey [26]. It examined the interaction of variables such as language spoken at home with the length of time a person has lived in Australia, and potential acculturation through employment and education. The results showed that the number of years lived in Australia was the crucial predictor of death literacy, rather than language at home. This reinforces the need to go beyond language translation for creating culturally responsive communication on EOL issues and services.

Although it is not uncommon in any culture for people to feel discomfort when speaking about death, in some cases, not talking about it is a cultural or religious norm: *the culture and idea for thousands of years passed on, for the Chinese, is privacy. Don’t bother other people. It’s my own suffering* (Mandarin FG). The research had representatives from both the Christian and Muslim faiths from Lebanon and Iraq, but also immigrants from Syria and Egypt. In the Arabic-speaking focus group, people of Muslim faith indicated that it was not part of their religious practice to speak about or plan for death.

That some migrant cultures may not directly speak about death and dying was (partially) reinforced by the DLI survey results [10]. Compared with Anglo Australians, migrants had significantly lower scores for the sub-scale asking how easy it would be to give support by talking to someone about death and dying. Nevertheless, such a conversation evidently occurred in some form, because the scores for ‘experience of discussing death and dying’ were the same for both Anglo Australians and migrants.

Difficult or traumatic histories can also limit migrants engaging in conversations about death and dying:

*Often when you have not grieved the first stress, or the first trauma, or the first grief, it goes underground. … They don’t even want to approach it because “if I unravel this thread, what rest will appear?”.*

(Indian counsellor, Community leader KI).

*We can’t manage their pain just with medication if they have psycho-social existential distress.*

(Clinical nurse consultant, Hospital palliative care unit KI).

Traditional cultural expectations may also dictate who is entitled to be cared for and who should do the caring at EOL. However, these expectations may clash with the need to interact with medical systems:

*Hindi speaking widows can believe themselves to be of such low worth that they would not be consenting to medical procedures for themselves on the basis of their own best interests.*

(Indian woman, Community leader KI).

*I do see quite a lot of ‘argy bargy’ within a family unit sometimes where, say, the wife might want to be the primary carer for the husband but the more educated or the more well to do person in the family might want to take over.*

(Clinical nurse consultant, Hospital palliative care unit KI).

Unlike a Western focus on individual consent, some cultures emphasise collective decision-making:

*It’s collective from the whole family* [his wife, children, and brothers]*. It takes into account mainly the person’s faith that they lived by, as well as their inherited norms and practices in their surrounding social environment.*

(Arabic FG).

While medical discourse focuses on the disease and symptoms of the patient, respondents highlighted the difference in priorities for migrant families whereby they want to be seen and known as a family unit with integrated roles and responsibilities:

*It was just not very understanding in terms of clinicians not being very empathetic, or culturally appropriate, or sensitive with the way they interacted with my family and in understanding the roles and responsibilities each of us play in my granddad’s life, and that was really important for us…no one [tried] to learn about the family dynamics.*

(Hindi PV).

In some cultures, the suggestion of administering palliative care can be interpreted as a betrayal of the dying person. For Arabic-speaking participants of Muslim faith, in contrast, strong medical interventions were an important sign of respect and affection: *we say that Allah the almighty, might grant him life again. So, they wait. Patience, for the end of a human being’s life is better.* (Arabic FG). 

Mandarin-speaking participants stated that their community had shifted toward focusing on a peaceful death: *in the past people thought we needed to let the person hang on to their last breath, that it was filial on the part of the children to see to that. Now, our thinking is changing, people now would like the person to go peacefully, to have peace in their final leg of the journey.* (Mandarin FG).

When hospital processes did not align with cultural traditions around care and rituals, participants expressed grief and guilt at not fulfilling cultural expectations:

*Until today I get nightmares …. In Chinese cultural traditions, when the body’s still really warm, we need to put on the formal best clothes for them to go. They [hospital staff] didn’t give us the opportunity to do all of that. …I can always see that bared, damaged back of my husband and I feel so sorry, so bad that he didn’t have the clothes on.*

(Mandarin PV).

Finding that it was *cultural* dimensions, rather than language differences, which caused the most stress is significant. This was also reflected in the WSLHD DLI survey results. The number of years in Australia was the most powerful demographic variable for predicting the DLI score [F(2,193) = 6.82; *p* < 0.001]; plus, there was a significant interaction because Arabic speakers of long-standing residence had very high DLI scores, unlike those who were recent migrants [F(4,193) = 3.15; *p* = 0.016]. Years in Australia was more important for acquiring death literacy than language background or whether the respondent spoke English at home, indicating that migrant access to EOL services was largely mediated by their familiarity with Australian culture and systems.

### 4.2. Strategies for Overcoming Barriers

#### 4.2.1. Making Greater Use of Existing Educational Resources

Multicultural Health (MCH) is a government service focusing on community education. At the time of our research, the educators had no specific programme for EOL and believed that communities would be reluctant to engage with such programmes. However, existing bilingual community educators could be upskilled to explain the available EOL services in culturally appropriate ways. Community educators would also know ways to approach the topic; for example, family caring is often a strong value which could provide context for a discussion of EOL services.

For many diseases, translations of educational materials are available in multiple languages, but these often reinforce the medical discourse around specific conditions. For EOL, resources providing guidance for families and loved ones was suggested, so they could *collect up all the info they need at end-of-life—account numbers, wills, family history, person’s end-of-life wishes,* etc. *This helps families be clear* (Death doula KI). Relevant print and electronic media with culturally appropriate language and images could be developed, but such material is best created in consultation with the relevant migrant communities to ensure cultural sensitivity.

However, as cultural humility suggests, ‘learning’ is not one-directional. While hospital health staff may bring knowledge for community leaders about EOL/palliative options, community leaders can bring valuable knowledge for staff about culture in EOL care:

*We’re learning from [the community] as much as they’re learning from us in regards to end-of-life care [services]…we ask… people what is it that we need to do for them to make sure their care is culturally acceptable. So, we’re [consulting] our patients for [our own] education.*

(Palliative care nursing manager KI).

Educational possibilities are also present in National and International days such as World Hospice and Palliative Care Day and Dying to Know Day (D2KD). These forums embody a wide variety of community events that encourage people to talk about their experiences. A clear CaLD presence would be a welcome addition. Further, multicultural events attended by a wide diversity of people could include stalls or tents with information about EOL support. These information booths may not be especially popular, but they could be welcomed by those with an immediate need and have the effect of normalising conversations where this would be helpful.

Reaching people, especially carers, who are in need of bereavement support is difficult because formal home services for the patient end at death. Some community centres provide informal gatherings for migrant groups which could be forums for (suitably trained) convenors and volunteers to identify people struggling with grief. They could then be linked to specialist services.

#### 4.2.2. Making the Most of Volunteers

Many hospitals have a formal volunteer programme with people trained to provide patient support. They provide culturally nuanced social interaction and help to bridge the various hospital silos. Often, the culture and languages of the volunteers will reflect those of the local communities:

*[Volunteers] come to it with quite a sense of responsibility for their community…if they meet anyone from their culture you know—it does touch them more.*

(Volunteer network KI).

Hospital health staff can also reflect the cultural diversity of the local area, and many are willing to provide useful assistance as informal volunteers. Bilingual medical staff are an important resource, such as *a resident [doctor] who does speak another language… because if we get a patient in from that language, you know we have direct communication with them* (Clinical nurse consultant, Hospital palliative care unit KI).

Generally, the volunteer role does not extend beyond the hospital, although some volunteers informally visit families at home. Nor does it address bereavement support, but it makes sense for a volunteer to continue supporting a family after the patient has died:

*They’ve come to volunteer to give back. So, they have an experience of a bereavement…It was always in my head after I set up the service to get palliative care volunteers into bereavement volunteer support. Because they may already have made links or because of…an interest in bereavement from their own experience in supporting people.*

(Volunteer network KI).

Formalising appropriately trained volunteers beyond the hospital would help to bridge the medical–bereavement divide. However, because many cultures rely on family and informal local networks for help, Western-style formal supervised volunteering may not be familiar, or trusted. It would therefore be useful to include this as a topic in education programmes.

Further suggestions for strengthening the volunteer service include: recruiting volunteers from diverse backgrounds; a register of formal and informal volunteers by language; training in cultural awareness and the role of palliative care; and integrating volunteers into the hospital team. A register of people and services for cultural community support (e.g., not-for-profit groups and religious leaders) could be especially useful.

#### 4.2.3. A Centralised Information Hub Bridging Existing Services

Bridging silos and making the most of existing services to streamline communication is an urgent need. A centralised information hub with one phone number and email address for accessing information and connecting with EOL and bereavement services would better support people at EOL, their carers, and families. This would include access to:

- Hospital services—palliative care, specialist units, occupational therapy and equipment, and volunteers and interpreters;
- Health services in the community—palliative care at home, community health, multicultural health, bilingual GPs and pharmacists, and bereavement counselling;
- Community services—community and spiritual leaders, psychosocial support groups, home help, and government agencies for aged care and pensions.

For each of these services, the languages spoken by bilingual staff would be recorded and the hubs themselves could provide a support team for each patient:

*If you had a team of a [culture specific worker], a [chronic and complex nurse] and a social worker… I think you need teams of the three. And those teams would be able to come together with network meetings to support each other*

(Drug and alcohol worker KI).

#### 4.2.4. Culturally Specific Supportive and Palliative Care Workers

The implementation of culturally specific positions in palliative care services could significantly help to bridge access barriers faced by migrant populations. Workers aligned to specific cultural groups with a knowledge of medical language and the health system and services would be ambitious in regions of high cultural diversity, such as WSLHD. However, this model is already in place and working well for Aboriginal communities in the LHD [10]. Their main role is communication facilitation, identifying new patients approaching EOL, and accompanying them in meetings with medical staff. They ensure that staff, patients and families all have a good understanding of what is happening and how their cultural needs can be met.

People in these positions supporting migrant needs would work across silos connecting within and between the hospital and community services. Developing a relationship of trust with patients and families would be crucial so they are well-placed to talk in an appropriate way about EOL care. With knowledge of the cultural groups’ experiences in the medical system, they can serve as advocates, advising the hospital and other services of people’s needs, including for bereavement.

#### 4.2.5. Making Hospital More Welcoming

There are some straightforward strategies that could be used throughout hospitals to make them more welcoming. Signs in a range of languages representing the local communities, and with images and symbols, not only communicate information but also give positive messages about diversity, which may feel welcoming. Quiet places for prayer or mediation need to be well sign-posted and within easy distance: *he didn’t have a proper place to pray…so that stopped him. Culturally…he felt…isolated* (Hindi PV). All groups expressed concern when visiting times were limited; for example, the Mandarin focus group noted:

*…ideally, [hospitals] could give the family more time to be with the patient … Like you can’t say ok you can spend time together, but it’s 8 o’clock now time to go. And if the patient really wants to have someone to talk to and yet there is nobody around to talk to, that’s just a terrible feeling, it’s just a lonely feeling.*

(Mandarin FG).

Participants emphasised the importance of staff taking time to develop trust and willingness to talk about difficult issues and checking that the patient has understood both the English and hospital jargon. Often, some acculturation to Western life has occurred [38,39] and there are variations among language groups, so it is important for staff to avoid making assumptions about patient preferences based on assumed cultural background: *it’s again checking in…because everyone can do things slightly differently and doesn’t mean they’re from the same community that they do things the same way* (Occupational therapist KI). Public hospitals are pressured, busy places and it is not easy for staff to slow down but poor communication creates more work, unnecessary distress, and poorer health outcomes. Indigenous awareness training is mandated in hospitals which could be extended to the needs of migrants.

In contrast to general hospital spaces, Palliative Care Units (PCUs) often have more flexibility. They have more time for relationship building that is about demonstrating respect for cultural needs, customs and beliefs, and the development of trust: *it’s about being present* (CNC Palliative Care KI) … *allowing them to get to know you, … and one day they will ask you those difficult questions, and they will trust you* (Palliative care nursing manager KI).

PCUs can have patient kitchen areas for the preparation/heating of appropriate food, *if the patient is in a position to eat anything [families] definitely don’t come empty handed…they come with food* (Hindi FG). Sufficient space for large gatherings around the patient is also needed so that family traditions and cultural practices can be maintained. Further, when the person dies, a room to sit with the deceased so they are not alone is also an important cultural need. Arabic focus group participants expressed that *visiting the patient is remuneration, reward, and mercy … and forgiveness* (Arabic FG).

To bridge barriers between PCUs and curative units of the hospital, palliative care specialist nurses would be a valuable resource in other units to help out and demonstrate good palliative care. This encourages other nurses to improve their care for dying patients and complete suitable records of their care:

*We have started using the Comfort Observation and Symptom Assessment … [we] identify a dying patient, and … [nurses] have to check as part of their 4-hourly obs.*

(Specialist palliative care nurse KI).

#### 4.2.6. Limitations of the Study

Three aspects identified here—location of study, language groups targeted, and population samples—potentially limit the generalisability of the findings. First, the study focused on a single local health district (LHD) with a large migrant population. Suggestions for service improvement in this location may not be applicable in places which have fewer government services, fewer migrants, or more accessible health systems with fewer bureaucratic processes for incoming migrants and refugees [10].

Second, research participation was limited to the three largest CaLD groups residing within the LHD. The target groups (Mandarin-, Hindi-, Arabic-speaking) are the second, third and seventh languages spoken worldwide [40], so they are significant populations and are reasonable to isolate for the scope of this study, given their prevalence in WSLHD. However, we acknowledge the many other language groups within the LHD and that each of the three target language groups could cover a diversity of speakers, dialects and cultural and religious mores.

Third, as is often the case with qualitative studies seeking specific and/or sensitive data, the population samples (KIs, FGs, PV) were recruited purposively (and to a lesser extent, via snowballing) rather than randomly and may not be representative samples. The purposive methods based on pre-existing relationships secured adequate qualitative samples, but the cohorts predominantly comprised those who had previous contact with MCH and WSLHD services. This marginalised the participation of people using private or community services outside the public health system.

Similarly, the DLI online survey attracted people who already had some interest in EOL issues. This led to higher scores for the target groups (Arabic-, Hindi- and Mandarin-speaking background migrants) than the general population (see [10]). The DLI result in the National Survey of 2019 [26] was 4.06, compared to 5.32 in the survey for this research.

Further/comparative research is implicated for generalizability across diverse populations. However, the study was funded by the NSW State Government specifically for WSLHD precisely because of its large migrant population and provides findings that may be helpful beyond that context, despite the potential limitations of location, target language groups and population samples.

## 5. Conclusions

The research revealed barriers to, and potential interventions for, ensuring access to formal health systems and community services to meet the EOL needs of migrant communities in WSLHD specifically, and in similar national and international contexts. Two broad responses are indicated. First, priority assistance for migrants to access the existing systems and services, while actioning improvements such as focused EOL education for staff and communities; greater use of volunteers; translators for services beyond hospitals; establishing information and drop-in centres; and having ‘system guides’ to help people to navigate and negotiate the complexity of services. This complexity is especially challenging for those who do not share the dominant culture within health systems.

Second, developing a long-term approach to EOL, and health more broadly, that is less Eurocentric and more open to diverse cultural perspectives. Siloing of services is a clear barrier yet is not institutionally essential. The Australian *National Palliative Care Standards* [41] require a palliative care philosophy that is able to meet the physical, cultural, spiritual, and psychosocial needs of individuals, families, and communities. However, this philosophy is not an easy fit with the dominant individualised health and welfare service discourse. Recognising this underlying discontinuity is a prerequisite for managers and their teams, driving institutional appropriate change to meet cultural needs in EOL service delivery.

Further, Compassionate Communities movements help whole communities, including health workers, recognise EOL as a social event in which medical treatment is just one component [21]. This approach is relevant to migrant communities where cultural traditions and practices at EOL can be facilitated at home, within the community and within formal health services. When the medical and social components combine smoothly, carers and patients can focus on physical and emotional comfort, making the path less distressing for the dying person. Dying is not one part of a mechanism breaking down. It is the final chapter in a rich human story. It is not a private choice. It is a universal human experience. Fostering this understanding, alongside cultural awareness, sensitivity, and humility, is an important part of improving services and support for migrant populations at EOL. Evidence from the Australian context provides substantial insight for policy makers and professionals aiming to ensure access to EOL and palliative care ‘for everyone, in every place, at every time’ (see [42,43,44]).

## Data Availability

The raw quantitative data (DLI Survey) supporting the conclusions of this article will be made available by the authors upon request. Restrictions apply to the qualitative datasets due to privacy criteria for consent on a topic that is highly sensitive for the participants and shared with researchers on the basis of trust.

## References

[1] Palliative Care Australia (PCA) What Is Palliative Care?. https://palliativecare.org.au/resource/what-is-palliative-care/.

[2] World Health Organization (WHO) Health Topics: Palliative Care. https://www.who.int/health-topics/palliative-care.

[3] United Nations (UN) (2015). Transforming Our World: The 2030 Agenda for Sustainable Development (A/RES/70/1).

[4] Abedini N.C., Downey L., Engelberg R.A., Curtis J.R., Sharma R.K. (2022). End-of-life healthcare utilization and palliative care use among older adults with limited English proficiency. J. Am. Geriatr. Soc..

[5] Six S., Bilsen J., Deschepper R. (2020). Dealing with cultural diversity in palliative care. BMJ Support. Palliat. Care.

[6] Barwise A.K., Nyquist C.A., Espinoza Suarez N.R., Jaramillo C., Thorsteinsdottir B., Gajic O., Wilson M.E. (2019). End-of-life decision-making for ICU patients with limited English proficiency. Crit. Care Med..

[7] Australian Institute of Health and Welfare (AIHW) (2016). 6.16 End-of-Life Care. Australia’s Health 2016.

[8] Horsfall D., Yardley A., Leonard R., Noonan K., Rosenberg J. (2015). End of Life at Home: Co-Creating an Ecology of Care.

[9] Rosenberg J.P., Horsfall D., Leonard R., Noonan K. (2018). Informal care networks’ views of palliative care services: Help or hindrance?. Death Stud..

[10] Leonard R., Paton J., Hinton P., Thomson J., Psychogios H. (2023). What Matters in the End: Understanding the End-of-Life Needs of Culturally & Linguistically Diverse and Aboriginal Communities in Western Sydney Local Health District.

[11] Fozdar F., Banki S. (2017). Settling refugees in Australia: Achievements and challenges. Int. J. Migr. Bord. Stud..

[12] Gunasekara A., Grant S., Rajendran D. (2019). Years since migration and wellbeing among Indian and Sri Lankan skilled migrants in Australia: Mediating effects of acculturation. Int. J. Intercult. Relat..

[13] Productivity Commission (2017). Introducing Competition and Informed User Choice into Human Services: Reforms to Human Services.

[14] Baker K., Adams J., Steel A. (2021). Experiences, perceptions and expectations of health services amongst marginalized populations in urban Australia: A meta-ethnographic review of the literature. Health Expect..

[15] Forrest J., Dunn K. (2007). Constructing Racism in Sydney, Australia’s Largest EthniCity. Urban Stud..

[16] Silva D.S. (2022). The abandonment of Australians in India: An analysis of the right of entry as a security right in the age of COVID-19. Monash Bioeth. Rev..

[17] Australian Bureau of Statistics (ABS) (2021). Snapshot of Australia: A Picture of the Economic, Social and Cultural Make-Up of Australia on Census Night. https://www.abs.gov.au/statistics/people/people-and-communities/snapshot-australia/2021.

[18] Aker N., Frost R., Walters K., West E., Davies N. (2023). Health inequalities for older people from minority ethnic groups receiving palliative care and end of life care: A scoping review protocol. PLoS ONE.

[19] Scanlon B., Brough M., Wyld D., Durham J. (2021). Equity across the cancer care continuum for culturally and linguistically diverse migrants living in Australia: A scoping review. Glob. Health.

[20] Sunvisson H., Habermann B., Weiss S., Benner P. (2009). Augmenting the Cartesian medical discourse with an understanding of the person’s lifeworld, lived body, life story and social identity. Nurs. Philos..

[21] Brown L., Walter T. (2014). Towards a social model of end-of-life care. Br. J. Soc. Work.

[22] Kellehear A. (2013). Compassionate communities: End-of-life care as everyone’s responsibility. QJM Int. J. Med..

[23] Karapliagkou A., Kellehear A. (2014). Public Health Approaches to End of Life Care: A Toolkit.

[24] Abel J., Kellehear A. (2016). Palliative care reimagined: A needed shift. BMJ Support. Palliat. Care.

[25] Noonan K., Horsfall D., Leonard R., Rosenberg J. (2016). Developing death literacy. Prog. Palliat. Care.

[26] Leonard R., Noonan K., Horsfall D., Kelly M., Rosenberg J.P., Grindrod A., Rumbold B., Rahn A. (2021). Developing a death literacy index. Death Stud..

[27] Department of Health, Disability and Ageing (2018). National Palliative Care Strategy 2018.

[28] Australian Institute of Health and Welfare (AIHW) (2025). Palliative Care Services in Australia. Web Report. https://www.aihw.gov.au/getmedia/d7994336-a4cf-42e5-8459-0edf50d5a706/Palliative-care-services-in-Australia.pdf?v=20251103170649&inline=true.

[29] Leonard R., Horsfall D., Rosenberg J., Noonan K. (2020). Carer experience of end-of-life service provision: A social network analysis. BMJ Support. Palliat. Care.

[30] Borgstrom E., Ellis J. (2017). Introduction: Researching death, dying and bereavement. Mortality.

[31] Caswell G., Turner N. (2020). Ethical challenges in researching and telling the stories of recently deceased people. Res. Ethics.

[32] Paton J., Horsfall D., Carrington A. (2018). Sensitive Inquiry in Mental Health: A Tripartite Approach. Int. J. Qual. Methods.

[33] Yeager K.A., Bauer-Wu S. (2013). Cultural humility: Essential foundation for clinical researchers. Appl. Nurs. Res..

[34] Kruger R.A., Casey M.A., Newcomer K.E., Hatry H.P., Wholey J.S. (2015). Focus Group Interviewing. Handbook of Practical Program Evaluation.

[35] Dewing J. (2008). Process Consent and Research with Older Persons Living with Dementia. Res. Ethics.

[36] Braun V., Clarke V. (2021). Thematic Analysis: A Practical Guide.

[37] Broom A., Good P., Kirby E., Lwin Z. (2013). Negotiating palliative care in the context of culturally and linguistically diverse patients. Intern. Med. J..

[38] Bosma H., Apland L., Kazanjian A. (2010). Review: Cultural conceptualizations of hospice palliative care: More similarities than differences. Palliat. Med..

[39] Lui C.-W., Ip D., Chui W.H. (2009). Ethnic experience of cancer: A qualitative study of Chinese-Australians in Brisbane, Queensland. Soc. Work Health Care.

[40] Statistics and Data. The Most Spoken Languages 2022. https://statisticsanddata.org/data/the-most-spoken-languages-2022/.

[41] Palliative Care Australia (PCA) (2018). National Palliative Care Standards.

[42] Gott M., Wiles J., Mason K., Moeke-Maxwell T. (2022). Creating ‘safe spaces’: A qualitative study to explore enablers and barriers to culturally safe end-of-life care. Palliat. Med..

[43] Gebauer S., Knox Morley S., Haozous E.A., Finlay E., Camarata C., Fahy B., FitzGerald E., Harlow K., Marr L. (2016). Palliative Care for American Indians and Alaska Natives: A Review of the Literature. J. Palliat. Med..

[44] Green A., Jerzmanowska N., Green M., Lobb E.A. (2018). ‘Death is difficult in any language’: A qualitative study of palliative care professionals’ experiences when providing end-of-life care to patients from culturally and linguistically diverse backgrounds. Palliat. Med..

